# Global Optimization of Ventricular Myocyte Model to Multi-Variable Objective Improves Predictions of Drug-Induced Torsades de Pointes

**DOI:** 10.3389/fphys.2017.01059

**Published:** 2017-12-19

**Authors:** Trine Krogh-Madsen, Anna F. Jacobson, Francis A. Ortega, David J. Christini

**Affiliations:** ^1^Greenberg Division of Cardiology, Department of Medicine, Weill Cornell Medicine, New York, NY, United States; ^2^Institute for Computational Biomedicine, Weill Cornell Medicine, New York, NY, United States; ^3^Cardiovascular Research Institute, Weill Cornell Medicine, New York, NY, United States; ^4^Physiology, Biophysics and Systems Biology Graduate Program, Weill Cornell Graduate School, New York, NY, United States

**Keywords:** cardiac modeling, model optimization, safety pharmacology, long QT, *in silico* drug trial, cardiotoxicity

## Abstract

*In silico* cardiac myocyte models present powerful tools for drug safety testing and for predicting phenotypical consequences of ion channel mutations, but their accuracy is sometimes limited. For example, several models describing human ventricular electrophysiology perform poorly when simulating effects of long QT mutations. Model optimization represents one way of obtaining models with stronger predictive power. Using a recent human ventricular myocyte model, we demonstrate that model optimization to clinical long QT data, in conjunction with physiologically-based bounds on intracellular calcium and sodium concentrations, better constrains model parameters. To determine if the model optimized to congenital long QT data better predicts risk of drug-induced long QT arrhythmogenesis, in particular Torsades de Pointes risk, we tested the optimized model against a database of known arrhythmogenic and non-arrhythmogenic ion channel blockers. When doing so, the optimized model provided an improved risk assessment. In particular, we demonstrate an elimination of false-positive outcomes generated by the baseline model, in which simulations of non-torsadogenic drugs, in particular verapamil, predict action potential prolongation. Our results underscore the importance of currents beyond those directly impacted by a drug block in determining torsadogenic risk. Our study also highlights the need for rich data in cardiac myocyte model optimization and substantiates such optimization as a method to generate models with higher accuracy of predictions of drug-induced cardiotoxicity.

## 1. Introduction

Mathematical models of cardiac electrophysiology are at the cusp of usage in a variety of clinical and pre-clinical applications, including safety pharmacology (Mirams et al., 2012; Zhang et al., 2016). In particular, mathematical modeling forms a central component in the Comprehensive *in Vitro* Proarrhythmia Assay (CiPA) initiative, a proposed strategy for progressing drug safety testing (Sager et al., 2014; Colatsky et al., 2016; Fermini et al., 2016; Gintant et al., 2016).

In terms of cardiotoxicity, drug safety testing aims to avoid Torsades de Pointes (TdP), a life-threatening ventricular tachycardia. Indeed, occurrences of drug-induced TdP in patients have lead to regulatory bans and market withdrawals of several drugs (Mirams et al., 2011). TdP risk is associated with prolongation of the QT interval on the electrocardiogram, in particular due to block of the hERG channel, which carries the rapid delayed rectifier current (I_Kr_; Sanguinetti et al., 1995; Straus et al., 2005; Hoffmann and Warner, 2006). However, multiple other currents and dynamics are of importance to torsadogenesis, and including measured effects of drugs on multiple channels, rather than just hERG, into TdP risk stratification improves risk prediction (Kramer et al., 2013; Mistry et al., 2015). Mechanistically, TdP initiation is linked to early afterdepolarizations (EADs) at the cellular level. Triggering of these EADs may depend directly on multiple different ionic currents, including the L-type calcium current (I_CaL_) and the late sodium current (I_NaL_) (Lankipalli et al., 2005; Hale et al., 2008), and may also depend on intracellular calcium and sodium dynamics (Terentyev et al., 2014; Kim et al., 2015; Xie et al., 2015; Krogh-Madsen and Christini, 2017), implying that the levels of the ionic transporters that control these concentrations (e.g., the sodium-calcium exchanger and the sodium-potassium pump) are important for torsadogenesis. Indeed, recent *in silico* work have pointed to the magnitudes of these two transporters as having large impact on TdP risk (Lancaster and Sobie, 2016).

Despite the proposed usage of mathematical models in safety pharmacology, even recent and sophisticated models of human ventricular myocyte electrophysiology perform poorly when simulating each of the most typical congenital long QT (LQT) syndromes (Mann et al., 2016). This naturally raises concerns about the ability of these *in silico* models to predict drug-induced LQT and TdP. However, using a global optimization strategy, *in silico* models can optimized to reproduce repolarization delays consistent with those seen clinically in the congenital LQT patient datasets, providing optimism for clinically-related model usage (Mann et al., 2016). A concern remains, however, as to whether these *in silico* models, optimized in terms of action potential properties, replicate dynamics of intracellular ionic concentrations well enough to reliably predict TdP risk. For example, when optimizing a model in terms of its electrical activity only, it can be difficult to correctly identify parameters that mainly control ionic concentrations (Groenendaal et al., 2015). Indeed, previous modeling studies have shown how identical-looking action potentials, modeled using different combinations of model parameters, can have differing calcium transients (Sarkar and Sobie, 2010).

To investigate this possible limitation, we therefore carried out a multi-variable optimization, using both clinical congenital LQT data and constraints on the concentrations on intracellular Ca^2+^ and intracellular Na^+^ ([Ca^2+^]_*i*_ and [Na^+^]_*i*_). We then asked whether optimized models that better represent the congenital LQT syndromes might allow for more accurate and more reliable modeling of acquired LQT and TdP risk. To this end, we simulated 86 cases of multi-channel drug block with known TdP risk level (Lancaster and Sobie, 2016) and found that the model optimized in terms of both action potential and [Ca^2+^]_*i*_, and [Na^+^]_*i*_ data, better predicts TdP risk.

## 2. Methods

### 2.1. Cell model and drug simulations

Simulations were performed using the O'Hara-Rudy (ORd; O'Hara et al., 2011) human ventricular ionic model as the baseline model, as this is the model proposed to be used in the CiPA initiative (Colatsky et al., 2016; Fermini et al., 2016). We used endocardial myocyte parameter settings except where otherwise noted (**Figure 4A**). We used a 1 Hz pacing rate and corresponding steady-state initial conditions (O'Hara et al., 2011). For each perturbation to the model (simulating drug block or LQT syndromes and parameter changes during the optimization; detailed below), the model was simulated for 500 beats prior to collecting data. We quantified action potential duration to 90% (APD_90_) or 50% (APD_50_) repolarization, as indicated. Calcium transients were characterized by diastolic level (the minimum [Ca^2+^]_*i*_ attained within an action potential cycle cycle) and systolic concentration (the peak [Ca^2+^]_*i*_ reached during an action potential). The [Na^+^]_*i*_ varies little within a single action potential and was measured as the maximum value.

For our drug simulations, we used the datasets of Kramer et al. (2013) and Mirams et al. (2011) as curated by Lancaster and Sobie (2016) with an associated yes/no risk of torsadogenesis. For each drug, the dataset gives its estimated effective free therapeutic plasma concentration (EFTPC), along with IC_50_ values for block of the channels generating I_Kr_, I_CaL_, and the fast sodium current (I_Na_). Drug effect on each channel type was modeled as a conductance block based on a Hill equation with a coefficient of 1:

(1) $$\begin{array}{r} G_{x,drug}=G_{x}{\left(1+\frac{\text{EFTPC}}{{\text{IC}}_{50,x}}\right)}^{-1}, \end{array}$$

where *G*_*x, drug*_ is the maximal conductance of channel *x* in the presence of the drug. The dataset contains 86 entries, with some duplicate drugs modeled differently by the two original sources. There are therefore 68 different compounds in the set, covering a variety of intended clinical use, including anti-arrhythmics, anti-histamines, antipsychotics, hypertension/angina drugs, and others.

### 2.2. Drug classification

To classify model output generated by these drug simulations we used a Support Vector Machine (SVM; Ben-Hur et al., 2008). We used linear decision boundaries separating two categories of data: TdP risk and no TdP risk. These decision boundaries were computed as the solution to a minimization of an error (E) calculated as the sum of squared distances between the location of miscategorized points and the boundary. Because the two variables used in the classification (APD_50_ and diastolic [Ca^2+^]_*i*_) have very different absolute values, we normalized them to baseline (i.e., no drug) values.

In general, the minimum value of E will take on different values when using different cell models to simulate drug effects, indicating that the separation of data points by category is better for some models than others. Therefore, to compare goodness of the classification between models and also to determine sensitivity of the decision boundaries, we calculate regions for which E remains below a threshold value (E^*^), which we set to twice the value of the lowest value of E found among the four tested models.

### 2.3. Model optimization

We optimized the baseline ORd model based on clinical data from LQT patients, following a similar strategy as Mann et al. (2016) and using their QT interval data for control patients and patients with one of the three most prevalent congenital LQT syndromes: LQT1, LQT2, or LQT3. The QT interval data from LQT1 and LQT2 patients came from a patient cohort with heterozygote nonsense mutations only, as that can be mimicked in the model by decreasing the conductances of I_Ks_ and I_Kr_ by 50%, respectively. The LQT3 cohort data is more heterogenous and the subtype was simulated by increasing the conductance of I_NaL_ by a factor that was allowed to vary as part of the optimization process. The amount of QT interval prolongation in these patient groups was 12.2% for LQT1, 16.6% for LQT2, and 16.2% for LQT3. Mapping the delayed repolarization measured clinically as QT interval prolongation directly to APD_90_ prolongation in the cell model, the objective data set was 267.97 ms (control), 301.14 ms (LQT1), 312.20 ms (LQT2), and 311.55 (LQT3).

In its simplest setup, the optimization was designed to minimize a sum-of-squares error from the APD_90_ objective when subjecting the model to control conditions and each of the LQT subtypes 1, 2, and 3. We refer to this as the “APD_LQT_" optimization. In other optimizations, we included [Ca^2+^]_*i*_ and [Na^+^]_*i*_ information in the objective to improve the optimization outcome. This “multi-variable” optimization was done by adding a hefty error (200 ms squared) if [Ca^2+^]_*i*_ and [Na^+^]_*i*_ fell outside a certain range during the control condition. We used a range of 0.05–0.15 μM for diastolic [Ca^2+^]_*i*_, 0.3–0.7 μM for systolic [Ca^2+^]_*i*_, and 7–10 mM for [Na^+^]_*i*_ based on measurements in human ventricular myocytes and recent modeling work (Beuckelmann et al., 1992; Piacentino et al., 2003; Grandi et al., 2010).

For the optimization method, we used a genetic algorithm (GA), which is a global optimization method that has been successful in optimizing cardiac ionic models to experimental and simulated data (Syed et al., 2005; Bot et al., 2012; Kaur et al., 2014; Groenendaal et al., 2015). We used a population size of 200 individual model instantiations and ran each GA for 50 generations. All other settings specific to the GA (detailing selection, crossover, mutation, and elitism) were defined as detailed previously (Bot et al., 2012). Because of the stochasticity inherent to the GA, each optimization was run ten times. We used the run resulting in the lowest error as the optimized model.

The parameters to be determined in the optimization process are scaling factors for the currents I_Kr_, I_CaL_, I_NaL_, the slow delayed rectifier current (I_Ks_), the sodium-calcium exchange current (I_NCX_), the sodium-potassium pump current (I_NaK_), and the extent of I_NaL_ increase with simulated LQT3. All scaling parameters were allowed to vary from 0.1% to 10-fold their values in the baseline model.

Note that for ease and consistency we will refer to current scaling factors as scaling of maximal conductances (and use G_Kr_, G_CaL_, G_NaL_, GKs, G_NCX_, and G_NaK_ for the currents defined above), although some currents are technically scaled by a permeability or a maximal charge carried.

## 3. Results

### 3.1. Sensitivity of APD, [Ca^2+^]_*i*_, and [Na^+^]_*i*_ in baseline model

To help guide our optimization procedure, we first did a sensitivity analysis to the major conductances as parameters with low sensitivity are problematic to determine in an optimization.

**Figure 1:**
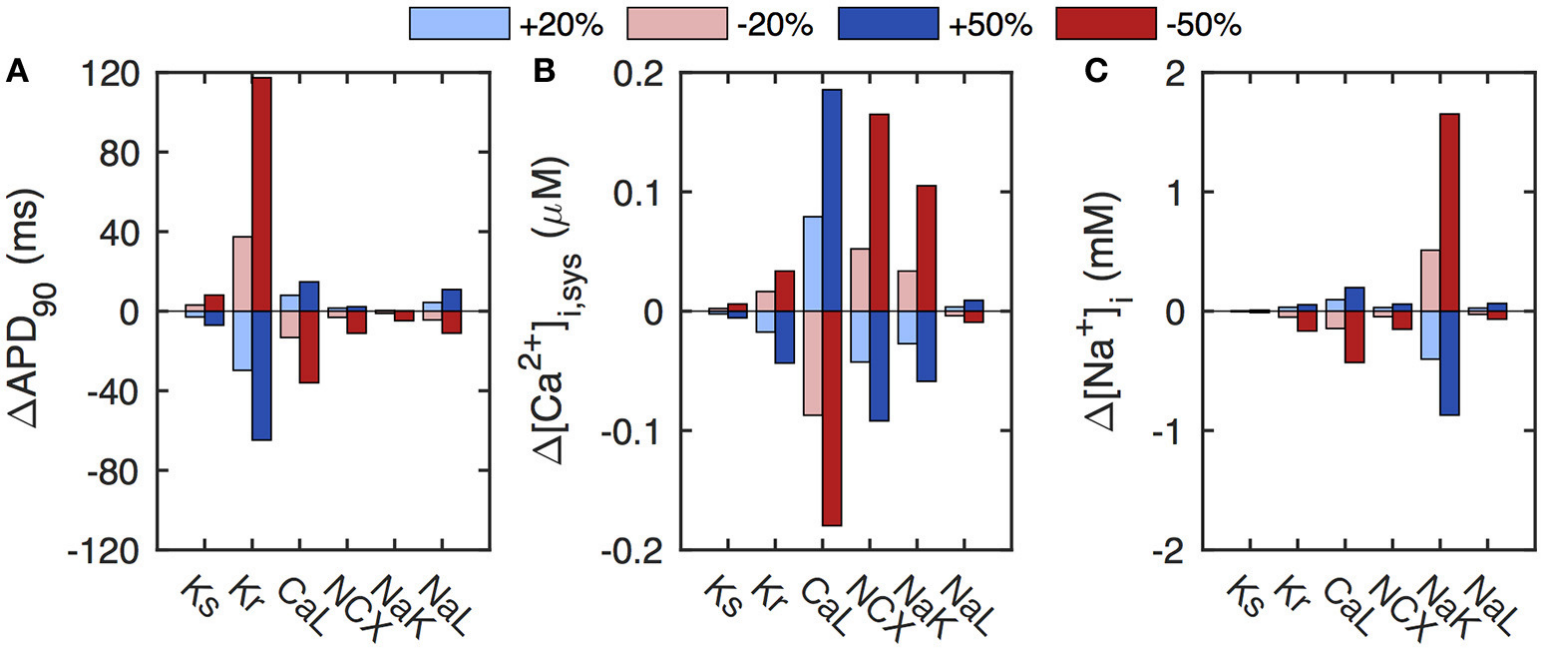
Sensitivity of baseline ORd model to select conductances. **(A)** The APD of the baseline model has a strong sensitivity to the conductance of I_Kr_. **(B)** The calcium transient (quantified here as systolic [Ca^2+^]_*i*_) depends sensitively on G_CaL_, G_NCX_, and G_NaK_. **(C)** The level of [Na^+^]_*i*_ depends mainly on G_NaK_. Conductances were varied by ±20% (light blue/red) and ±50% (dark blue/red) of baseline values.

In the baseline ORd model, the action potential duration of the ORd model is highly sensitive to changes in I_Kr_ (Figure 1A). For example, when decreasing G_Kr_ by 50% to simulate LQT2, the response is a prolongation of APD_90_ by 117 ms (44%), substantially larger than the QT interval prolongation of 68 ms (16.6%) seen in LQT2 patients with heterozygote nonsense mutations (Mann et al., 2016). The APD has an intermediate sensitivity on G_CaL_, but shows little sensitivity to variations in GKs and G_NaL_, the currents associated with LQT1 and LQT3, respectively. For example, reducing GKs by 50% to mimic LQT1, gives a modest 8-ms (3%) APD_90_ prolongation, much shorter than the 51 ms (12%) QT interval prolongation seen clinically (Mann et al., 2016).

As expected, the calcium transient has a very different parameter sensitivity dependence. It depends strongly on the conductances of I_CaL_ and I_NCX_, with a 50% increase in G_CaL_ or a 50% reduction of G_NCX_ increasing systolic [Ca^2+^]_*i*_ by almost 0.2 μM (Figure 1B). The calcium dynamics also has a significant dependence on G_NaK_, which only controls [Ca^2+^]_*i*_ indirectly via [Na^+^]_*i*_ changes that regulate I_NCX_. Indeed, [Na^+^]_*i*_ depends sensitively on G_NaK_, with a 50% reduction in G_NaK_ resulting in a 1.7 mM increase in [Na^+^]_*i*_ (Figure 1C). Variations in the remaining key conductances have little influence on [Na^+^]_*i*_ levels.

These results are consistent with those presented previously for ±10 and ±20% parameter variations in the ORd model (O'Hara et al., 2011).

### 3.2. Model optimization

As it is difficult to estimate parameters to which an output is not sensitive, the above analysis suggests that if optimizing the baseline ORd model to APD data only, it will be problematic to estimate many of the conductance parameters. Including repolarization delay data from LQT types 1, 2, and 3 as additional information to the objective may help determine the scaling of GKs, G_Kr_, and G_NaL_. Further, pinpointing these parameters may narrow down other conductances that correlate with these more directly determined parameters (Groenendaal et al., 2015). The sensitivity analysis also indicates that inclusion of calcium transient data to the optimization objective should help determine G_NCX_ and G_NaK_ scaling, and that incorporation of [Na^+^]_*i*_ may further help determination of G_NaK_ scaling.

**Figure 2:**
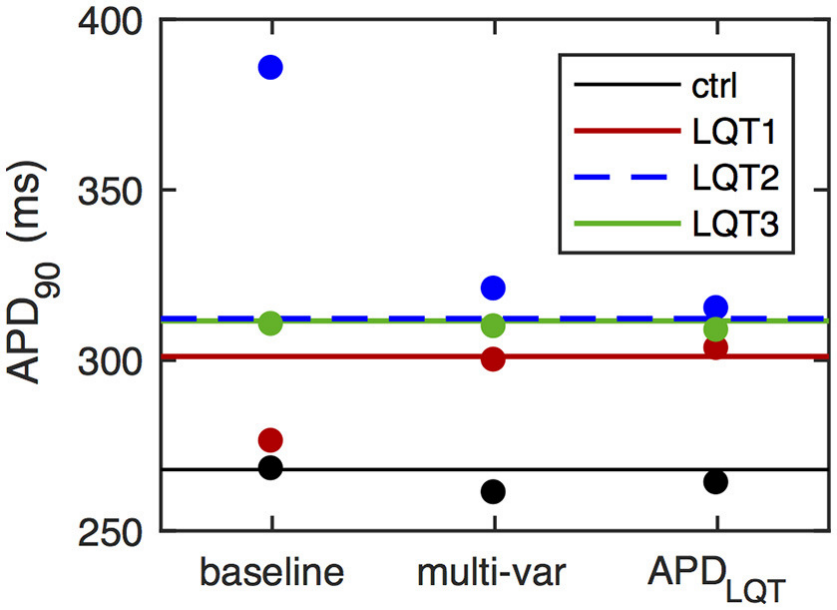
APD values of optimized models. Horizontal lines give control APD_90_ (black) as well as APD_90_ surrogates for QT interval prolongation in LQT patients (colored). These APD values form the optimization objective in the simplest case (“APD_LQT_”). For the multi-variable optimization (“multi-var”), the objective also include constraints on [Ca^2+^]_*i*_ and [Na^+^]_*i*_. Dots give APD_90_ values under control simulations (black) and during LQT simulations (LQT1, red; LQT2, blue; LQT3, green). The overestimation of the LQT2 response and the underestimation of the LQT1 response in the baseline ORd model are eliminated in the optimized models. Relative APD_90_ prolongation in the baseline model is 3.0, 43.8, and 15.8%, for LQT1, LQT2, and LQT3, respectively. For the multi-variable optimized model, relative APD_90_ prolongation is 14.9, 22.9, and 18.6% for LQT1-3, while for the APD_LQT_-optimized model the corresponding values are 14.5, 19.4, and 17.0%. The target QT interval values are 12.2, 16.6, and 16.2%.

We therefore optimized the baseline ORd model to both clinical QT interval data from LQT patients and [Ca^2+^]_*i*_ and [Na^+^]_*i*_ as detailed in section 2.3. The model optimized to this multi-variable objective produces APD_90_ values that are within 3% of their target values (Figure 2). The optimized parameter scaling factors are given in Table 1. The optimized model has a much increased GKs, resulting in a larger response to the simulated LQT1 condition, matching the target data (Figure 2).

**Table 1:** Scaling factors for optimized models.

**Objective**	**Ks**	**Kr**	**CaL**	**NCX**	**NaK**	**NaL**	**NaL_LQT3_**
Multi-variable	8.09	1.17	3.57	3.05	1.91	1.70	4.17
APD_LQT_	9.71	1.42	9.59	1.75	7.40	4.86	2.28

*The optimization gave estimated parameters for scaling of the currents I_Ks_, I_Kr_, I_CaL_, I_NCX_, I_NaK_, and I_NaL_, and for the increase of I_NaL_ during simulated LQT3. Optimizing to APD values only (APD_LQT_) resulted in a very different parameter set compared to the multi-variable optimization that include of [Ca^2+^]_i_ and [Na^+^]_i_ constraints. In particular, it resulted in much increased scalings for I_CaL_ and I_NaK_*.

Optimizing the baseline model to APD values only (i.e., omitting the [Ca^2+^]_*i*_ and [Na^+^]_*i*_ constraints) results in slightly better matching of the objective (Figure 2; errors with 2%). Optimized parameter values are very different, with large increases in scaling of G_CaL_ and G_NaK_ in addition to the enhanced GKs scaling (Table 1).

**Figure 3:**
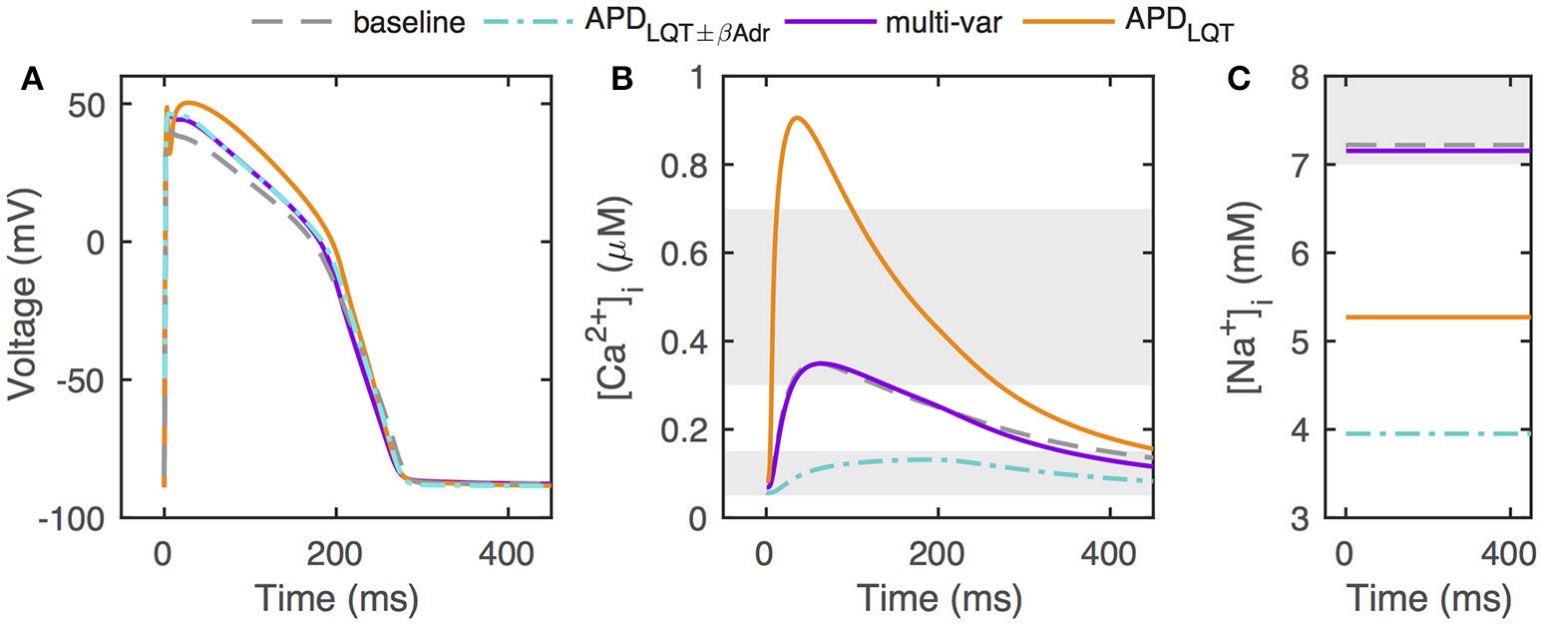
Action potentials, calcium transients, and [Na^+^]_*i*_ in optimized models. **(A)** Optimized models have similar action potentials under control conditions, but the different parameter sets underlying the different solutions give rise to some waveform variation. **(B)** Despite having comparable action potentials, models optimized without constraints on [Ca^2+^]_*i*_ and [Na^+^]_*i*_ can have widely different calcium transients. Shaded areas give constraints on minimum and maximum [Ca^2+^]_*i*_ (0.05–0.15 and 0.3–0.7μM, respectively). **(C)** Without constraints on [Ca^2+^]_*i*_ and [Na^+^]_*i*_, optimization can result in models with very low [Na^+^]_*i*_ levels. Shaded area indicate constraint on [Na^+^]_*i*_ (7–10mM). “APD_LQT±βAdr_” designates the original optimization to clinical LQT data under normal and β-adrenergic stimulation conditions by Mann et al. (2016).

Despite the diversity in parameter scalings among the baseline and the optimized models, the action potential morphology is quite similar across these differently parameterized models (Figure 3A). We also include for comparison the action potential generated by Mann et al. in an optimization to clinical LQT data under both baseline and β-adrenergic conditions (“APD_LQT±βAdr_” optimization, Mann et al., 2016). The main difference among the action potential waveforms is a depolarization of phase 2 of the action potential, the amount of which correlates with the upscaling of G_CaL_ from the baseline model (about 2–4 in the multivariable and APD_LQT±βAdr_ models, and almost 10 in the APD_LQT_ model).

However, calcium transients and [Na^+^]_*i*_ levels are vastly different across models (Figures 3B,C). When including [Ca^2+^]_*i*_ and [Na^+^]_*i*_ constraints in the optimization, the optimized calcium transient and [Na^+^]_*i*_ level are very close to those of the baseline model, despite the allowed ranges being relatively large (0.05–0.15 μM for diastolic [Ca^2+^]_*i*_, 0.3–0.7 μM for systolic [Ca^2+^]_*i*_, and 7–10 mM for [Na^+^]_*i*_). In our optimizations, when optimizing to APD only, the calcium transient is significantly enhanced. This is consistent with the much boosted G_CaL_ and the decreased G_NCX_ scaling (relative to the multi-variable optimization) both of which favor a larger calcium transient. In addition, the G_NaK_ scaling is much increased when optimizing to APD only, consistent with the lower [Na^+^]_*i*_. For the APD_LQT±βAdr_ optimized model (Mann et al., 2016), both G_NCX_ and G_NaK_ were much increased relative to baseline (scaling of 2.95 and 9.12, respectively), resulting in a small-amplitude calcium transient and a low [Na^+^]_*i*_.

### 3.3. TdP prediction

**Figure 4:**
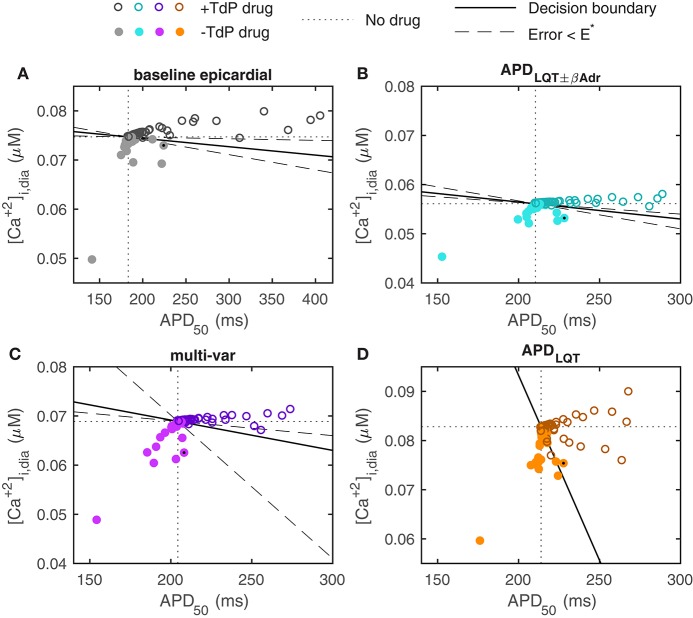
Prediction of TdP risk from model simulations. **(A)** Baseline ORd model with epicardial myocyte parameter settings (the epicardial configuration is shown here as it was determined to give the best classification in Lancaster and Sobie, 2016. Using the endocardial baseline model yields very similar results). **(B)** APD_LQT±βAdr_ optimized model. **(C)** Multi-variable optimized model. **(D)** APD_LQT_ optimized model. Dotted lines indicate no-drug control values of APD_50_ and diastolic [Ca^2+^]_*i*_. Colors for the different models correspond to the color scheme in Figure 3. Solid lines give decision boundaries between torsadogenic (open circles) and non-torsadogenic drugs (filled circles). Dashed lines demarcate regions within which the categorization error remains below a threshold value (E^*^). Using the multi-variable optimized model, all drugs that prolong APD_50_ by more than 5 ms are known TdP risk drugs. Verapamil (marked by black dot) is an example of a TdP negative drug that significantly prolongs the AP in the baseline and APD-optimized models but not in the multi-variable optimized model.

To test how well the optimized models predict TdP risk, we used a dataset consisting of drugs blocking I_Kr_, I_CaL_, and I_Na_ to varying degrees, and their associated risk of torsadogenesis (TdP positive or TdP negative; Lancaster and Sobie, 2016). As demonstrated previously, while many of the drugs in this dataset that carry a TdP risk do prolong APD, some drug simulations predict an increased APD for TdP negative drugs (Figure 4A, Lancaster and Sobie, 2016). In particular, in the baseline model, simulations of three non-torsadogenic drugs results in action potential prolongation of 15–25 ms (one of these is noted by a black dot in Figure 4A). As demonstrated by Lancaster and Sobie, simulations of those three drugs also led to a decreased diastolic [Ca^2+^]_*i*_, which was not seen in the TdP positive drugs. Therefore, including diastolic [Ca^2+^]_*i*_ as a second metric (APD_50_ being the first) by which to classify the drugs, allows for a correct TdP risk categorization of these three otherwise false positives (Figure 4A, Lancaster and Sobie, 2016). Indeed, using APD_50_ and diastolic [Ca^2+^]_*i*_ in combination correctly classifies drugs in the dataset with high specificity and sensitivity (Figure 4A, Lancaster and Sobie, 2016).

For the APD_LQT±βAdr_ optimized model (Mann et al., 2016), qualitatively similar results are observed (Figure 4B). The particular values of diastolic [Ca^2+^]_*i*_ over which TdP positive drugs are separated from TdP negative drugs are shifted, reflecting baseline differences. The same three TdP negative compounds that resulted in APD prolongation in the baseline model, give increased APD_50_ in this optimized model as well.

When simulating drug application in the multi-variable optimized model, predictions are improved (Figure 4C). In particular, none of the TdP negative drugs result in APD_50_ prolongation beyond 5 ms, implying that APD_50_ prolongation in itself is a strong predictor of torsadogenic risk in this model. In addition, many of the TdP negative compounds result in more substantial reductions in APD_50_ and/or diastolic [Ca^2+^]_*i*_ compared to the baseline and the APD_LQT±βAdr_ optimized models. There is therefore an increased flexibility to the positioning of the decision boundary separating the TdP positive from the TdP negative drugs (dashed lines in Figure 4C mark off area within which the categorization error remains less than the threshold value, E^*^).

For the APD_LQT_ optimized model, the classification is less successful (Figure 4D). The same three TdP negative drugs that resulted in false positive APD prolongation in the baseline and in the APD_LQT±βAdr_ optimized model do so here. Further, simulation of several TdP positive drugs result in decreased diastolic [Ca^2+^]_*i*_ without much change in APD and therefore form false negatives in this categorization. The presence of these false positives and negatives pose a challenge to the classification and prevents the categorization error from getting below the threshold value regardless of the location of the decision boundary.

What are the ionic mechanisms underlying the improvement in predictive ability by the multi-var model? The simulated drugs that give the false positive APD prolongations for the baseline and APD-optimized models are piperacillin and verapamil (note that two independent measurements for verapamil are included in the dataset). These drugs block both I_CaL_ and I_Kr_. We investigated the ionics of verapamil (black dots in Figure 4) in more detail, as verapamil is a well-known example of an I_Kr_-blocking agent that does not prolong the QT-interval and is not torsadogenic (Redfern et al., 2003).

**Figure 5:**
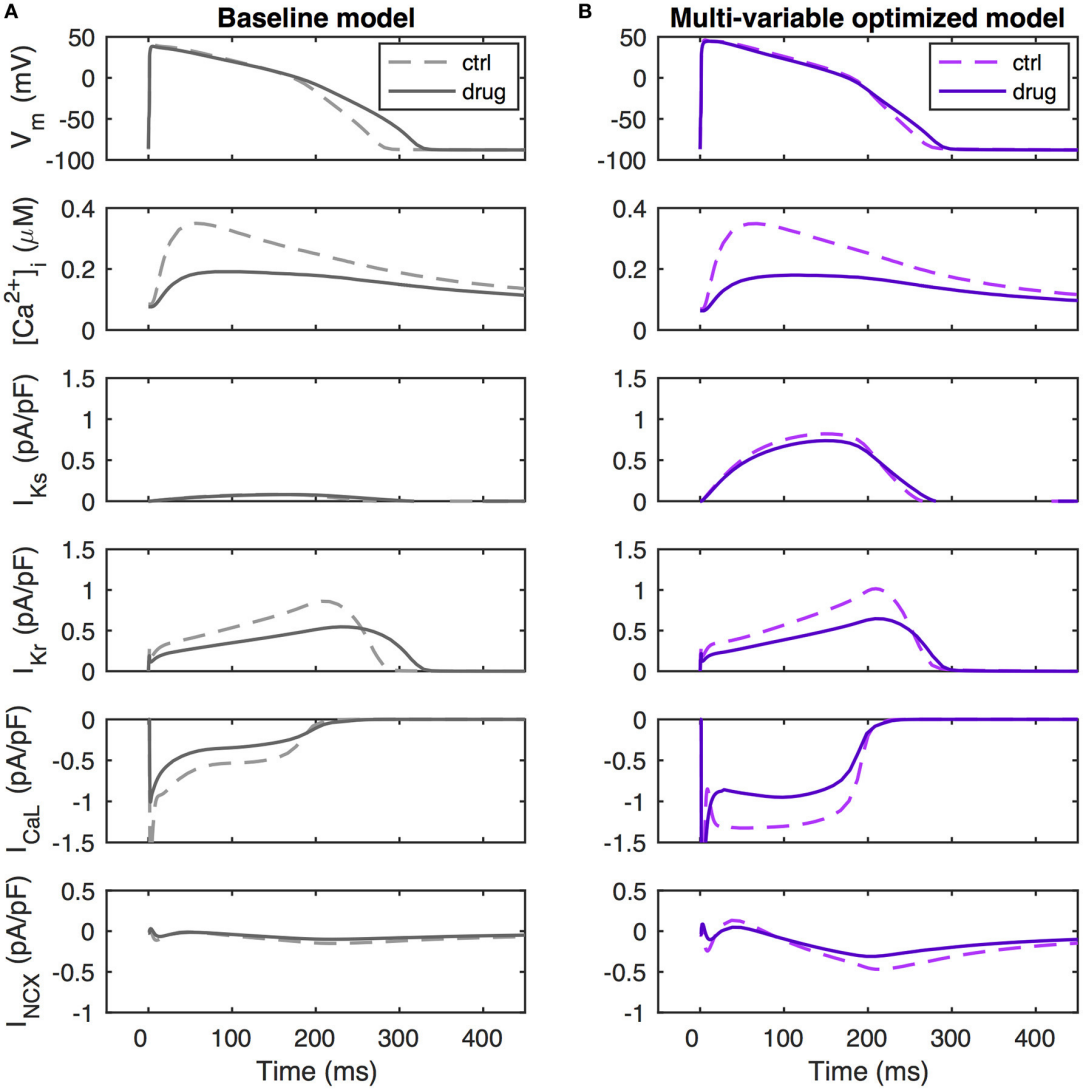
Ionic mechanisms of repolarization dynamics during verapamil application. **(A)** Baseline model. **(B)** Multi-variable optimized model. Verapamil (simulated as a scaling of G_CaL_ by 0.64, a scaling of G_Kr_ by 0.55, and a scaling of G_Na_ by 0.998) decrease I_Kr_ by similar amounts in the baseline and in the multi-variable optimized models. However, due to the up-regulated I_CaL_ and I_NCX_ in the optimized model, it sustains a larger loss of inward current than the baseline model. Further, the increased I_Ks_ in this model provides a repolarization reserve. Together, these effects lead to a maintained APD_50_ value and an only slightly increased value of APD_90_.

A simulation of verapamil application in the baseline model is shown in Figure 5A. Drug-induced reductions in outward I_Kr_ and inward I_CaL_ are seen to be of similar amplitude and balance each other during the first 200 ms of the action potential. When I_CaL_ inactivates at this time, the loss of outward I_Kr_ is largely unopposed, leading to a decreased rate of repolarization and APD prolongation. In the multi-variable optimized model, the non-drug action potential is generated through a near-balance between a much increased I_CaL_ and a larger I_NCX_ providing inward current against the outward currents I_Kr_ and I_Ks_, with I_Ks_ now being of similar size as I_Kr_ (Figure 5B). When simulating verapamil application in the multi-var optimized model, there is a loss of inward current by the direct effect of I_CaL_ conductance block and because of a reduction of I_NCX_ due to the decreased calcium transient. As both I_CaL_ and I_NCX_ are increased in the multi-variable optimized model relative to the baseline model, the loss of inward current with verapamil application is amplified, preventing repolarization delays. Further, the increased I_Ks_ in the multi-var model helps maintain repolarization under verapamil application. Thus, factors beyond the scaling of the directly blocked currents I_Kr_ and I_CaL_ contribute to the drug-induced response.

## 4. Discussion

We investigated optimization of conductance parameters in a human ventricular myocyte model to match clinical data from LQT patients using constraints on the concentrations of intracellular calcium and sodium ions. Without these constraints, parameter optimization can lead to models with unphysiological calcium transient and [Na^+^]_*i*_. To test the hypothesis that the optimization would allow the model to make improved predictions of drug-induced arrhythmogenesis, we investigated the ability of the model to determine TdP risk in a large set of known drugs. We found that using the optimized model improves TdP prediction in two complementary manners. First, simulations of three TdP negative drugs that result in APD prolongation using the baseline model result in no or minimal APD prolongation when using the optimized model. Second, when using both diastolic [Ca^2+^]_*i*_ and APD_50_ for the model-based drug classification, the optimized model gives an improved separation between the TdP positive and negative drugs, measured as an increased flexibility in the positioning of the decision boundary. Based on these findings, our main conclusion is that intracellular ionic concentrations are important for safety pharmacology modeling.

### 4.1. *In silico* TdP prediction

Other studies have investigated ionic-model-based TdP prediction using different approaches. It is clear from these studies that a range of strategies can be applied to improve TdP prediction. First, there is improvement to the baseline model, which in itself can involve a number of approaches. One is to optimize a model to clinical LQT data, as done here or previously (Mann et al., 2016). Conceptually, fitting a cellular model to clinical ECG data rather than to experimental cellular-level data may appear counter-intuitive, but it makes sense given that the model is used to predict an organ-level, rather than a cellular-level, arrhythmia risk. Another model optimization approach is to tune the model to experimental data obtained with ion channel blockers. Such data can be additional to the data used originally to build the baseline model, as in the example of a canine model that upon optimization delivered improved prediction of test drug data (Davies et al., 2012). In another article in this Research Topic, Dutta et al. (2017) used drug data presented in the original ORd model paper to reparameterize the ORd model. This optimization was done in conjunction with another model improvement strategy: re-casting the I_Kr_ description as a Markov model with state- and voltage-dependent drug block (Li et al., 2017). An alternative strategy to optimizing a model is to generate a population of models to represent inter-individual and/or inter-cell variability, potentially recapitulating variability in drug response across a heterogeneous population (Lancaster and Sobie, 2016; Britton et al., 2017). Another contribution to this Research Topic demonstrates that such population models predict TdP risk better than using a single baseline model (Passini et al., 2017). Population models may also be used to gain mechanistic insights into arrhythmogenesis. For example, Passini et al. determined that different sub-populations of models had different propensities to repolarization abnormalities, with low conductances for the outward currents I_Kr_ and I_NaK_ and increased levels of I_CaL_ and I_NCX_ making models more prone to repolarization abnormalities, emphasizing that currents other than I_Kr_ are important in this aspect.

Second, TdP prediction may be improved by selection of better risk measures. While repolarization delay (APD or QT interval prolongation) has high sensitivity to TdP positive drugs, its specificity is more limited, with some drugs prolonging QT interval, yet carrying only low TdP risk (e.g., amiodarone; Sager et al., 2014). Measures that may be useful in risk stratification include diastolic [Ca^2+^]_*i*_ (Lancaster and Sobie, 2016) as employed here. While this measure was selected, in combination with APD_50_, from a range of action potential and calcium transient biomarkers through a machine learning process, there is a mechanistic basis as to why [Ca^2+^]_*i*_ levels may be associated with TdP risk, as abnormal intracellular calcium dynamics and spontaneous calcium release is associated with EAD formation, a cellular-level trigger of TdP (Lancaster and Sobie, 2016; Němec et al., 2016). Another risk measure proposed from *in silico* work is the net charge carried during the action potential by six major ionic currents (Dutta et al., 2017; Li et al., 2017). This measure may also be mechanistically linked to TdP arrhythmogenesis, as it is indicative of robustness against EAD generation under a G_Kr_-reduction challenge (Dutta et al., 2017). Use of repolarization abnormality occurrence (i.e., EADs or incomplete repolarization) in simulations as a metric for TdP risk may also present a viable stratification pathway (Passini et al., 2017). Given the direct link to arrhythmogenesis, this seems like a promising risk marker, but a possible limitation lies in its use of highly elevated drug concentrations to trigger the repolarization abnormalities, which may lead to an overestimation of the number of false negatives. Use of this metric rather than APD prolongation improves TdP prediction in a population of models, but not in the baseline ORd model (Passini et al., 2017).

In summary, it is clear that *in silico* cell models can be improved to better predict TdP risk and that measures beyond APD prolongation are helpful to this end, but it also apparent that significant uncertainties remain as to how to best carry out the modeling and the arrhythmia risk prediction.

### 4.2. Kr/Ks balance

Our optimization resulted in significant rescaling of many parameters, in particular GKs, which was increased approximately eight-fold. This is comparable to the scaling of 5.75 found in Mann et al. (2016). In the baseline ORd model, I_Ks_ is relatively small under control conditions, its peak value during an action potential being roughly 10 times smaller than peak I_Kr_. Significant upscaling of this current is therefore necessary to recapitulate the clinical LQT1 phenotype showing substantial QT interval prolongation with loss of I_Ks_. Likewise, the Grandi-Bers model, another recent human ventricular myocyte model (Grandi et al., 2010) that has little reliance on I_Ks_ under control conditions, requires sizeable upscaling of GKs (about 25-fold) to reproduce the LQT1 clinical data (Mann et al., 2016). In contrast, the ten Tusscher-Panfilov human ventricular myocyte model (ten Tusscher and Panfilov, 2006) which has similarly sized I_Ks_ and I_Kr_, requires increased G_Kr_ (2.65-fold) and decreased GKs (0.41-fold) to reproduce the clinical LQT dataset (Mann et al., 2016).

These substantial increases in GKs required for the ORd and the Grandi-Bers models to reproduce the clinical LQT data are at odds with the I_Ks_ ranges recorded experimentally. Factors that may contribute to this disagreement include: (1) Differences between levels of β-adrenergic-dependent kinases and phosphorylation which regulate I_Ks_ and exacerbate LQT1 (Wu et al., 2016); (2) Transmural or other intra-heart heterogeneity with some regions having especially delayed repolarization; (3) Methodological experimental limitations with I_Ks_ rundown and/or damage of the I_Ks_ channel due to enzymatic digestion—however, the recordings that formed the basis of GKs in the ORd model were done in small tissue preparations using microelectrodes for the express purpose of mitigating these complications (O'Hara et al., 2011).

The I_Ks_ conductance was also increased (by 87%) in the recent optimization of the ORd model with the Markov I_Kr_ formulation to the original O'Hara et al. data (Dutta et al., 2017). While this approximately doubled I_Ks_ at baseline, I_Ks_ remained much smaller than I_Kr_ (by about five-fold) and would not be expected to be able to reproduce the LQT1 phenotype. Because the particular balance between I_Kr_ and I_Ks_ can be important for action potential stability and EAD generation (Devenyi et al., 2017), one may expect a model with large GKs to behave differently from a model with smaller GKs model in terms of arrhythmogenesis. Because the reasons for the discrepancy between the experimental and the clinical I_Ks_ data are not known, it will likely be useful to the field to have both a model with large GKs, replicating the clinical LQT data, and a model with smaller GKs, replicating the experimental data.

The mismatch between the experimental and the clinically-based estimations of GKs also raises broader questions regarding how to best handle inconsistent data in model development. Our approach here has been to use data of perceived highest relevance to the particular type of predictions made, i.e., to use clinically-based parameter estimations to predict clinical arrhythmia risk. This approach is in line with the general strategy of using data specific to a particular system (e.g., a cell or a patient) to generate a model specific to that system. However, the best way forward may be to couple rigorously uncertainty in model parameters to uncertainty in model predictions using uncertainty quantification tools (Johnstone et al., 2016).

### 4.3. Effect of verapamil on action potential duration

The balance between different currents is also important for determining a model's response to simulated drug block. The anti-hypertension and anti-angina drug verapamil blocks I_CaL_ and I_Kr_, does not prolong the QT interval, and does not prolong APD in recordings from human trabeculae (Redfern et al., 2003; Britton et al., 2017). However, different human *in silico* models give different responses to simulated verapamil application. The Grandi-Bers and the ten Tusscher-Panfilov models predict action potential shortening in response to verapamil (Mirams et al., 2011, 2012). In variations of the ORd model, verapamil almost always prolongs the APD, but the response varies depending on drug concentration, on how block is modeled, and on whether a Markov model is used for I_Kr_ (Britton et al., 2017; Dutta et al., 2017; Passini et al., 2017).

One hypothesis as to why verapamil does not prolong APD is that its block of I_Kr_ is compensated for by block of I_CaL_. Using our multi-var optimized model, we show here that in addition to the block of I_CaL_, a secondary reduction in I_NCX_ (due to the decreased calcium transient) is important in off-setting the I_Kr_ block by verapamil. The size of the I_Ks_ current is also important in determining APD under I_Kr_ block conditions as I_Ks_ provides a repolarization reserve. However, I_Ks_ level in itself is not predictive of APD shortening with verapamil since in the APD_LQT_ optimized model, which has a much increased repolarization reserve in I_Ks_, verapamil leads to APD prolongation.

### 4.4. Limitations

There are several limitations to our modeling and optimization approach. We allowed large ranges of the scaling (0.1% to 10-fold) of the parameters to be estimated in the optimization. Consequentially, the conductance scalings may be unphysiologically large, with, e.g., GKs becoming larger than estimated experimentally. However, we are explicitly not attempting to make the best model of a single cell or small tissue, but, rather, a model capable of making clinically relevant predictions. We did not include the clinical data from control and LQT types 1, 2, and 3 patients during β-adrenergic stimulation (Mann et al., 2016) in the optimization objective as preliminary optimizations with this addition resulted in adrenergically stimulated action potentials having unsmooth repolarization profiles, characterized by slow late repolarization. Due to experimental difficulties in determining absolute values of [Ca^2+^]_*i*_ and [Na^+^]_*i*_, we based the allowed ranges of these mainly on modeling work, particularly the ORd and the Grandi-Bers models. While experimental measurements of [Ca^2+^]_*i*_ in human ventricular myocytes are consistent with the simulated values (Beuckelmann et al., 1992; Piacentino et al., 2003), reported measurements of [Na^+^]_*i*_ are much higher (~20 mM), but may be overestimated (Pieske et al., 2002; Grandi et al., 2010). While the inclusion of bounds on [Ca^2+^]_*i*_ and [Na^+^]_*i*_ provided additional information to constrain conductance parameters, it is likely that inclusion of more data into the objective would help constrain parameters further. Such data could include more repolarization markers, further calcium transient features, and drug block data.

We modeled the drug application using a simple conductance block, although some drugs are known to block in a state-dependent manner (Mirams et al., 2011; Di Veroli et al., 2014; Britton et al., 2017; Dutta et al., 2017). However, use of this simpler approach allowed us to simulate a larger drug data set. We used a single model for the drug simulations. It might be valuable in future work to generate a population of models around the optimized model to potentially improve predictions and to give insights into ionic mechanisms underlying population heterogeneity in drug responses.

## Author contributions

TK-M: Designed study, carried out simulations, and analyzed data; AFJ and FAO: Implemented the optimization software. All authors contributed to critical revision of the manuscript.

### Conflict of interest statement

The authors declare that the research was conducted in the absence of any commercial or financial relationships that could be construed as a potential conflict of interest.
